# A New Insight to Bone Turnover: Role of **ω**-3 Polyunsaturated Fatty Acids

**DOI:** 10.1155/2013/589641

**Published:** 2013-11-04

**Authors:** Naroa Kajarabille, Javier Díaz-Castro, Silvia Hijano, Magdalena López-Frías, Inmaculada López-Aliaga, Julio J. Ochoa

**Affiliations:** ^1^Institute of Nutrition and Food Technology “José Mataix Verdú”, Biomedical Research Center, Health Sciences Technological Park, University of Granada, Avenida del Conocimiento s/n, Armilla, 18071 Granada, Spain; ^2^Departament of Physiology, Faculty of Pharmacy, Campus de Cartuja s/n, University of Granada, 18071 Granada, Spain

## Abstract

*Background*. Evidence has shown that long-chain polyunsaturated fatty acids (LCPUFA), especially the **ω**-3 fatty acids such as eicosapentaenoic acid (EPA) and docosahexaenoic acid (DHA) are beneficial for bone health and turnover. *Objectives*. This review summarizes findings from both *in vivo* and *in vitro* studies and the effects of LC PUFA on bone metabolism, as well as the relationship with the oxidative stress, the inflammatory process, and obesity. *Results*. Some studies in humans indicate that LCPUFA can increase bone formation, affect peak bone mass in adolescents, and reduce bone loss. However, the cellular mechanisms of action of the LCPUFA are complex and involve modulation of fatty acid metabolites such as prostaglandins, resolvins and protectins, several signaling pathways, cytokines, and growth factors, although in certain aspects there is still some controversy. LCPUFA affect receptor activator of nuclear factor **κ** **β** (RANK), a receptor found on the osteoclast, causing bone resorption, which controls osteoclast formation. *Conclusions*. Since fatty acids are an endogenous source of reactive oxygen species, free radicals alter the process of bone turnover; however, although there are clinical evidences linking bone metabolism and dietary lipids, more clinical trials are necessary to prove whether **ω**-3 PUFA supplementation plays a major role in bone health.

## 1. Introduction

Omega-3 polyunsaturated fatty acids (*ω*-3 PUFA) are a group of essential fatty acids (FA), and they cannot be synthesized in sufficient amounts for health, therefore, they are components of the human diet [1]. Alpha-linolenic acid (ALA), eicosapentaenoic acid (EPA), and docosahexaenoic acid (DHA) are three important omega-3 fatty acids. ALA is the omega-3 fatty acid ingested in greatest amount in a typical diet and is found in leafy vegetables, walnuts, soybeans, and seed and vegetable oils. Sources of EPA and DHA are fatty fish such as salmon, fish oil supplements, or the conversion of ingested alpha-linolenic acid to DHA or EPA, although evidence reports that the conversion rate is low [2]. EPA and DHA have many potential health benefits, reducing risk of coronary heart disease [3], and potential benefits in the prevention and treatment of other cardiovascular disorders, some forms of mental illness, inflammatory disorders, and insulin resistance [1, 4].

Hence, it has been suggested that *ω*-3 PUFA play an important role in bone metabolism and may represent a useful nonpharmacological way of ameliorating bone loss and risk of osteoporosis. *ω*-3 is precursor for several potent regulatory eicosanoids involved in bone metabolism including PG and leukotrienes. Thereby, *ω*-3 PUFA can inhibit the production of these inflammatory cytokines such as IL-1, IL-6, and TNF-*α*, which provide an important stimulus for osteoclastic bone resorption, and suppression of the production of these cytokines by *ω*-3 PUFA may inhibit bone resorption and prevent bone loss [5].

To date few studies have examined the role of these essential fatty acids, particularly *ω*-3 PUFA in skeletal metabolism in humans, and the findings show controversy [6]. One possible explanation for these conflicting observations is that *ω*-3 PUFA have relatively modest effects on bone metabolism, making it difficult to detect changes after supplementation, and this may be a particular issue for indices such as bone mass density (BMD) which are relatively insensitive to short-term change as compared with biochemical bone turnover markers [7]. The differences in *ω*-3 PUFA supplementation also may be crucial: *α*-linolenic acids like EPA and DHA or all *ω*-3 PUFA in general are thought to have many different roles and actions in the body, which may or may not be linked to skeletal metabolism. *ω*-3 PUFA dose, supplement intake, and duration may also have different effects [8].

## 2. Bone Metabolism

Bone remodeling is regulated by controlling osteoblast (bone-forming cells) and osteoclast (bone-resorption cells) cell number and activity. Osteoclastogenesis is in large part regulated by a triad of proteins consisting of a ligand, receptor-activated nuclear kappa-*β* ligand (RANKL), its receptor, RANK, and a decoy receptor, osteoprotegerin (OPG). RANK is a membrane-bound protein expressed on osteoclast precursors and mature osteoclasts [9]. RANK-L exists in both a membrane-bound and soluble form and is produced by a range of cells including osteoblasts and activated T cells. OPG is a soluble protein secreted by different cells, including osteoblasts. Binding of RANK-L to RANK leads to osteoclastogenesis and inhibits osteoclast apoptosis [10]. Binding of RANK-L to OPG prevents RANK-L/RANK-induced osteoclastogenesis, and increased OPG protein levels promote a rapid reduction in osteoclast number. Moreover, the balance between RANK, RANK-L, and OPG is a major factor controlling osteoclast number [8]. 

Bone remodeling occurs within the skeleton and is activated in response to mechanical strain. Osteocytes are “mechanosensing” specialized cells that reside in bone matrix. They detect mechanical strain and initiate signaling pathways, promoting both osteoclastogenesis and osteoblastogenesis [8]. The role of lipid mediators in the signaling pathway is critical. In few seconds after mechanical loading of bone, the lipid mediator prostaglandin E2 (PGE2) is released by osteocytes and mature osteoblasts [11]. Phospholipase-mediated membrane releases fatty acids; mainly arachidonic acid (AA, 20:4n-6), the substrate for PGE2 synthesis, and expression of the inducible form of cyclooxygenase (COX), COX-2, which oxidizes AA to PGE2, are upregulated as an early response [12]. PGE2 promotes osteoclastogenesis by stimulating expression of both RANK-L and RANK and inhibiting expression of OPG. PGE2 also activates the Wnt signaling pathway and promotes core binding factor *α*-1 (cbfa-1) and insulin-like growth factor 1 (IGF-1) expression, thus stimulating osteoblastogenesis [13]. PGE2 is potent modulators of bone remodeling, affecting bone resorption, by stimulating osteoclastogenesis through RANKL. Both processes, formation and resorption, are related to PGE2, and its effects on bone may be dose-dependent. At high levels, PGE2 suppresses osteoblast and promotes differentiation and bone resorption by osteoclasts via RANKL, whereas low levels stimulate bone formation by osteoblasts [14].

Although the importance of AA and PGE2 in regulating bone remodeling is well established, the involvement of LCPUFA in the control of bone metabolism may be much more extensive than being currently known.

## 3. Physiological Factors Affecting Bone Metabolism 

### 3.1. Oxidative Stress and Bone Remodeling

Reactive oxygen species (ROS) are oxygen-containing molecules, which are produced during normal metabolism. Overproduction of ROS induces an imbalance between antioxidants and prooxidants in cells and tissues causing oxidative stress, which has been related to ageing, tissue inflammation, and degeneration [15]. 

According to evidence, there is a link between bone metabolism and redox balance regulation, indicating that ROS may play a major role in osteoporosis in part by inhibiting osteoblast generation. ROS are also involved in cartilage homeostasis and degradation, in particular with respect to the development of osteoarthritis (OA). Rheumatoid arthritis (RA) is an autoimmune disease characterized by chronic inflammation which causes joint destruction. Although, RA is not generally recognized as a disease of oxidative stress, it has been suggested that the level of ROS in patients with RA is higher than in healthy subjects. Researches have also reported that ROS could be a key modulator of bone cell function and play a role mediating intracellular signaling [16]. Thus, the ultimate mechanism behind ROS contribution to bone metabolism is still controversial.

Physical activity plays role in bone health; in this way, it is recommended as a treatment for the prevention of bone loss. Exercise influences the cellular homeostasis, and some *in vitro* studies have reported that mechanical loading increased the oxidative stress in chondrocytres and osteoblast-like cells. In contrast, exercise can lead to an increase in some antioxidants in bone as well as cartilage [17, 18].

During normal physiological conditions ROS are produced at low levels and removed by endogenous antioxidant systems. Their “steady-state” concentrations are determined by the balance between their rates of production and removal by various antioxidants [15]. Mitochondria are considered as the main source of intracellular ROS, but other enzymatic systems, such as NADPH oxidases, cytochrome P-450, cyclo-oxygenase, aldehyde oxidase, dihydroorotate dehydrogenase, tryptophan dioxygenase, nitric oxide synthase, and xanthine oxidase, contribute also to ROS production. Furthermore, both aging and oestrogen deficiency increase the generation of ROS, and there is evidence to suggest that adverse effects of oestrogen loss on bone may be prevented by antioxidants. Hence, ROS are also produced in response to external stimuli, such as growth factors, inflammatory cytokines, chemotherapeutics, environmental toxins, ultraviolet light, or ionizing radiation [18].

Enzymatic antioxidant defenses include superoxide dismutase (SOD), catalase (CAT), glutathione peroxidase (GPx), and glutathione reductase (GR). These can be modified by exercise, nutrition, and aging. Nonenzymatic antioxidants include a variety of quenchers such as ascorbic acid, *α*-tocopherol, carotenoids, flavonoids, thiols which include glutathione (GSH), ubiquinone Q 10, uric acid, bilirubin, ferritin, and micronutrients which act as enzymatic cofactors [18].

At the molecular level, cellular stress response pathways are controlled by four categories of molecules and transcriptional regulators: insulin/IGF-1 signalling, sirtuins, target of rapamycin, and AMP-activated protein kinase-dependent pathways. All these pathways have one molecular target in common, named forkhead box O (FoxO) transcription factors, a family of transcription factors that comprises four members: FoxO1/FKHR, FoxO3/FKHRL1, FoxO4/AFX, and FoxO6. FoxOs play an important role in bone biology by enabling the maintenance of a physiologically appropriate lifespan of mature osteoblasts through their defense activities or via modulating the activity of other transcription factors such as *β*-catenin [19]. The antioxidant defense provided by FOxOs is overtaken by high oxidative stress and/or oxidative stress activated pathways that interfere with the activity of FoxOs. ROS play a double role as both deleterious and beneficial species, because they can be either harmful or beneficial to living systems, particularly by playing a physiological role in intracellular signalling and regulation as secondary messengers. Nevertheless, signalling via ROS is harmful as overproduction of reactive signal molecules may be destructive. A large variety of diseases are known to involve oxidative stress, including bone disease [18].

**Figure 1:**
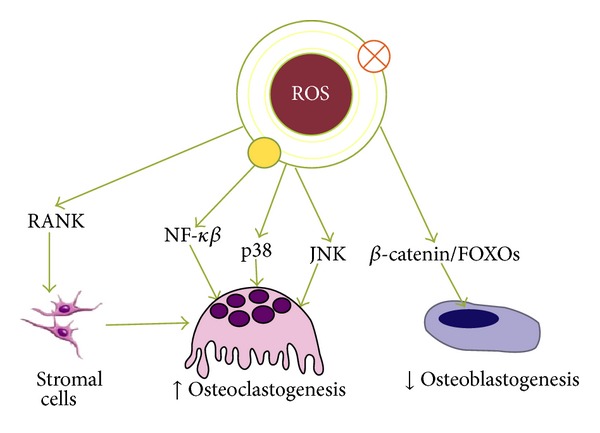
ROS-activated signalling pathways affecting the genesis of osteoblasts and osteoclasts. In osteoclast precursors, RANKL-induced activation of RANK stimulates ROS production, which is important for osteoclastogenesis. ROS-induced bone resorption occurs through the modulation of kinases and transcription factor activities in both osteoclasts and osteoblasts.

Bone remodeling is performed by osteoblasts and osteoclasts. Tipping this balance in favour of the osteoclasts leads to pathological bone resorption, as seen in bone diseases. ROS are involved in apoptosis of osteoblasts and osteocytes and osteoclastogenesis and in this way in bone resorption [20]. Actually, ROS have a spectrum of responses ranging from proliferation to growth or differentiation arrest to cell death by activating numerous signalling pathways, heat shock factor (HSF), and MAPKs including extracellular signal-regulated kinases (ERKs), c-Jun-N terminal kinase (JNK), p38 MAPK, and ERK5. The osteoprotegerin (OPG), the receptor activator of NF-*κβ* (RANK), and the receptor activator of NF-*κβ* ligand (RANKL) play also a crucial role in bone remodeling and functions as a pivotal molecular link for osteoblast and osteoclast coupling [21] (Figure 1). 

Mitochondria and ROS, particularly H_2_O_2_, play a crucial role in osteoclast function and differentiation. ROS increases osteoclast number and resorption by stimulating RANKL and TNF-*α* expression through ERK and NF-*κβ* activation. TNF-*α* not only causes cell damage but also inhibits SOD1 and SOD3 [22]. 

RANKL activates mature osteoclasts and mediates osteoclastogenesis. It binds to its receptor, RANK, promoting their differentiation into mature osteoclasts. OPG acts as a decoy receptor for RANKL, avoiding it from binding to and activating RANK. Abnormalities of the RANK-RANKL-OPG system with an unbalanced increase in RANKL activity have been implicated in the pathogenesis of various skeletal diseases, including osteoporosis and bone disease secondary inflammation. The increased osteoclastic activity may increase the superoxide anion (O_2_^−•^) generation and/or inhibit SOD and GPx activities with concomitant bone destruction [23].

Osteoblasts can produce antioxidants to protect against ROS, such as GPx, as well as transforming growth factor-*β* (TGF-*β*), which is implicated in reduction of bone resorption. Bai et al. (2005) [21] have found that H_2_O_2_ increased intracellular ROS and suppressed the differentiation process of osteoblasts, manifested by a reduction of differentiation markers including type 1 collagen, ALP, and colony-forming unit-osteoprogenitor (CFU-O) formation. H_2_O_2_ or xanthine/xanthine oxidase-induced oxidative stress is also able to inhibit bone cell differentiation of preosteoblastic cell line (MC3T3-E1) and of a marrow stromal cell line (M210B4) that undergoes osteoblastic differentiation. Oestrogens antagonize ROS-induced osteoblast apoptosis and the prosurvival effects of receptor activator of RANKL on osteoclasts and decrease oxidative stress. It has been suggested that elucidation of these mechanisms provides a paradigm shift from the “oestrogen-centric” account of the pathogenesis of involutional osteoporosis to one in which age-related mechanisms intrinsic to bone are central to the disease process [18, 24].

In response to stress, DNA-damage repair genes that counteract the adverse effects of ROS and FoxOs upregulate enzymatic scavengers, therefore representing an indispensable homeostatic mechanism for skeletal health. This response involves upregulation of the expression of the mitochondrial enzyme SOD2, that converts hydroxyl radicals into H_2_O_2_, the peroxidase catalase, which converts H_2_O_2_ into water, and the growth arrest and DNA-damage-inducible GADD45 [25]. Furthermore, FoxOs may control the generation of new osteoblasts from their mesenchymal stem cell progenitors by modulating their proliferation and/or differentiation through their antioxidant properties. Some studies have found a diminution in antioxidant activity in patients with osteoporosis. GPx1, the enzyme primarily responsible for the intracellular degradation of H_2_O_2_, is highly expressed in osteoclasts, and its expression in bone marrow macrophages by RANKL and in osteoclasts by oestrogens. Overexpression of GPx in osteoclast progenitors abolishes osteoclast formation and suppresses RANKL-induced NF-*κβ* activation and increased resistance to oxidation [26]. Oxidative stress acted as an independent risk factor for osteoporosis linked to the increase of SOD/GPX ratio. Hence, the imbalance propitiated by greater SOD activity with respect to GPx promotes the increase in H_2_O_2_ levels, leading to greater oxidative stress [18]. 

### 3.2. Inflammation and Bone Remodeling

Human studies have reported wide evidence to implicate the crucial role of osteoclasts in the pathogenesis of bone erosions in patients with rheumatoid arthritis (RA). The role of osteoclasts in bone resorption has been found to be also crucial in other inflammatory arthritis. 

Ritchlin et al. (2003) [27] have found that patients with psoriatic arthritis, who have characteristically elevated serum TNF-*α* levels, have a significant increase in the osteoclast precursor cell pool within the peripheral blood mononuclear cells populations that in turn are correlated with the extent of bone destruction. The fact that osteoclasts are largely responsible for focal bone erosions has increased efforts in the researchers to elucidate the exact role played by a number of cytokines and inflammatory mediators that has the capacity to induce the recruitment, differentiation, and activation of these osteoclast [28].

RANKL plays a role in controlling osteoclastogenesis. Inflamed synovial tissue produces a variety of other cytokines and hormones that can also influence osteoclastogenic activity. These factors include interleukin-1*α* (IL-1*α*), IL-1*β*, TNF-*α*, IL-6, macrophage colony stimulating factor (M-CSF), IL-17, and parathyroid hormone related hormone related peptide (PTHrP) [28]. 

The role of these cytokines in bone erosions and in inflammation reports further evidence to a link between immune system activation and bone resorption. When the function of OPG/RANK/RANKL in bone remodeling was perceived, it was hypothesized that RANKL may be of major pathophysiological importance in the bone and joint destruction observed in inflammatory RA [29]. 

**Figure 2:**
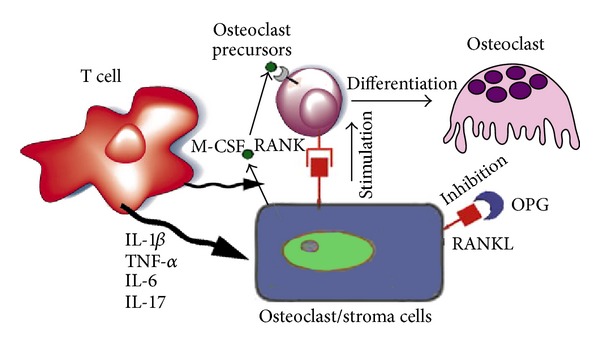
The OPG/RANK/RANKL system and osteoclastogenesis.

According to some studies, activated T cells from the RA synovium and synovial fibroblasts produce RANKL [29]. Indeed it is currently known that activated T cells, which play a central role in the pathogenesis of RA, may contribute to the osteoclast-mediated bone resorption via RANKL expression (Figure 2).

In bone destruction associated with RA, IL-17-producing helper T cells (TH17) play a major role by inducing receptor activator of nuclear factor-*κβ* ligand (RANKL). RANKL stimulates osteoclastogenesis through nuclear factor of activated T-cells cytoplasmic 1 (NFATc1), which is a crucial regulator of immune response [30]. In addition to cellular interactions via cytokines, the immune and skeletal systems share various molecular pathways, including membrane receptors, signaling molecules, and transcription factors.

### 3.3. Obesity and Bone Remodeling

The relationship between obesity and bone is complex and continues being an active research area. Current data from epidemiological and animal studies have shown that fat accumulation may interfere with bone mass. Obesity possibly affects bone metabolism through several mechanisms. Both adipocytes and osteoblasts are derived from a common multipotential mesenchymal stem cell, thereby obesity may increase adipocyte differentiation and fat accumulation while decreasing osteoblast differentiation and bone formation. Obesity is also in relation to chronic inflammation. The increased circulating and tissue proinflammatory cytokines in obesity may stimulate osteoclast activity and bone resorption through modifying the receptor activator of NF-*κβ* (RANK)/RANK ligand/osteoprotegerin pathway. Moreover, the excessive secretion of leptin and/or decreased production of adiponectin by adipocytes in obesity may either directly affect bone formation or indirectly affect bone resorption through upregulated proinflammatory cytokine production. High-fat intake may be detrimental to intestinal calcium absorption and therefore decrease calcium availability for bone formation [31].

A growing evidence supports that obesity and bone metabolism are linked. Firstly, both osteoblasts (bone forming cells) and adipocytes (energy storage cells) are derived from a common mesenchymal stem cell and agents inhibiting adipogenesis stimulated osteoblast differentiation [32] and vice versa, those inhibiting osteoblastogenesis increased adipogenesis. Second, decreased bone marrow osteoblastogenesis with aging is usually accompanied with increased marrow adipogenesis. Third, chronic use of steroid hormone, such as glucocorticoid, leads to obesity accompanied by rapid bone loss. And fourth, both obesity and osteoporosis are associated with increased production of proinflammatory cytokines and elevated oxidative stress [33]. 

Obesity is traditionally thought to be beneficial to bone and, hence protecting against osteoporosis. Mechanical loading stimulates bone formation by decreasing apoptosis and increasing proliferation and differentiation of osteoblasts and osteocytes through the Wnt/*β*-catenin signaling pathway [34]. But excessive fat mass may not protect from osteoporosis and in fact, and increased fat mass is associated with low total bone mineral density and total bone mineral content [31].

According to available literature to date, obesity seems to affect bone metabolism through several mechanisms. Obesity may decrease bone formation (osteoblastogenesis) while increasing adipogenesis because adipocyte and osteoblasts are derived from a common multipotential mesenchymal stem cell. While research with obese animal model has established the negative effects of adiposity on bone metabolism, studies with human subjects continue to be controversial [31].

Obesity may increase bone resorption through upregulating proinflammatory cytokines such as IL-6 and TNF-*α*. These proinflammatory cytokines are capable of stimulating osteoclast activity through the regulation of the RANKL/RANK/OPG pathway. Obesity is in relation to degenerative and inflammatory musculoskeletal system. Bone marrow adipocytes may also directly regulate the osteoclast progenitors (hematopoietic cells) [31, 35]. 

Obesity appears to affect bone metabolism directly or indirectly through adipocyte-derived cytokines such as leptin and adiponectin. Obesity is linked with significant increase in serum leptin and decrease in adiponectin. The action of leptin on bone may be complex and both positive and negative effects have been reported [36]. Its action seems to depend on current leptin status and the mode of the action. Obese subjects have low serum adiponectin concentrations as compared to those normal subjects. Increased secretion of leptin (and/or decreased production of adiponectin) by adipocytes may also contribute to macrophage accumulation by simulating transport of macrophages to adipose tissue and promoting adhesion of macrophages to endothelial cells, respectively [37].

Based on current data obesity is detrimental to bone health. The decreased bone mass with obesity may be due to increased marrow adipogenesis at the expense of osteoblastogenesis, increased osteoclastogenesis due to the upregulation of proinflammatory cytokines, excessive leptin secretion or reduced adiponectin production [31].

In fact, a high-fat diet, a common cause of obesity, has been reported to interfere with intestinal calcium absorption. Free fatty acids can form unabsorbable calcium soaps and contribute to low calcium absorption. Increased body weight associated with obesity may counteract the detrimental effects of obesity on bone metabolism. It is well known that body weight or body mass index (BMI) is positively correlated with bone mineral density or bone mass [38] and low body weight or BMI is a risk factor for low bone mass and increased bone loss in humans. Although some studies indicate that the positive effects of body weight could not completely compensate the detrimental effects of obesity on bone, at least in obese animal models [31].

## 4. Nutrition in Bone Remodeling Process 

Diet can be modified for the maintenance and development of bone mass. The most relevant nutrients to bone health are calcium and phosphorus because they compose roughly 80% to 90% of the mineral content of bone hydroxyapatite; protein is essential because it is incorporated into the organic matrix of bone for collagen structure upon which mineralization occurs; and other minerals, trace elements, and vitamins (e.g., vitamins D and K) are also crucial in carrying out metabolic processes and reactions in bone [29, 30]. Other benefits on bone metabolism derives from bioactive components found basically in vegetables but also some herbs and fruit. Phytochemicals, antioxidants, and other bioactive compounds influence bone metabolism through a variety of mechanisms manly in reducing oxidative stress and inflammation [37].

Data from the Framingham study [31] concluded that participants who had a diet based on fruit, vegetables, milk, and cereals had a significantly higher bone mass than those whose diet was characterized by high consumption of snacks, pizza, and/or high consumption of meat, bread, and potatoes. Nakashima and Takayanagi [30] evaluated associations between dietary patterns and bone mineral density (BMD) in Japanese farmwomen and found that a “healthy” pattern, described by high intakes of green and dark yellow vegetables, mushrooms, fish and shellfish, and fruit, was positively related to BMD. Furthermore, the effect of vegetarian dietary patterns on skeletal health has been studied; lacto-ovo vegetarians appear to have normal bone mass when compared with omnivores, whereas a more recent review reported that the scientific findings support the hypothesis that vegans do have lower BMD than their nonvegan counterparts [29, 32]. 

An adherence to a Mediterranean diet pattern is not associated with indices of bone mass (in a sample of adult women), whereas adherence to a dietary pattern close to the Mediterranean diet, that is, high consumption in plant foods and olive oil, low in meat and dairy products, and with moderate intake of alcohol, is positively related to bone mass and total body bone mineral content, suggesting potential bone-preserving properties of this pattern throughout adult life [29]. 

It is known that the intake of fruit and vegetables is positively associated with bone health by provision of essential micronutrients for bone formation, buffer precursors which reduce acid load, and phytochemicals affecting inflammation and oxidative stress.

The beneficial effect is because of the provision of micronutrients magnesium, potassium, calcium, vitamins A, C, E, and K, and potentially a lower dietary acid load conferred by the fruit and vegetables food group [33]. Typical diets are acidic because predominantly acid (hydrogen ions) rather than base (bicarbonate) is created during the metabolism of the daily food intake. Acid forming grains and high protein food derived from animal origin (meat, fish, and eggs) contain sulphur-based amino acids, methionine, and cysteine which create acid when metabolized. Alkaline forming foods contain potassium salts which can be broken down to make alkaline buffers [34]. Vegetables and fruit are considered alkaline because of their high mineral content in the form of salts of organic acids. The salts, predominantly potassium based but also calcium and magnesium, generate bicarbonate to balance the acid produced from the rest of the diet [35].

The low grade metabolic acidosis induced by the modern diet is exacerbated during ageing when renal function begins to decline requiring the body's skeletal reserves to be called upon to relinquish bicarbonate to produce alkaline buffers needed to continuously balance the acid load. Therefore bone mass is gradually and indefinitely reduced after the age of 30 years, accelerating at menopause to lower bone strength and mineral density. Fruit/vegetables influence on acid-base balance is crucial, being the unique dietary source of alkaline precursor constituents, so it is important reason to increase consumption to avoid bone loss during ageing [35, 36].

An increase in vegetable and fruit consumption to ≥9 servings/day will reduce the estimated net endogenous acid production by approximately 20 mEq/day and result in reduction in bone markers of resorption C telopeptide of type 1 collagen (CTx) and bone formation marker Procollagen 1 N-terminal peptide (P1NP) in postmenopausal women. Those women who include 4-5 servings of vegetables, herbs, and fruit featured bone resorption inhibitory properties (BRIPs) and half of the 9 servings/day will reduce resorption marker CTx by a greater amount. It is also hypothesised that this increase in fruit and vegetable intake will significantly affect inflammatory and metabolic markers including C-reactive protein (CRP) adiponectin, interleukin 6 (IL-6), interleukin 10 (IL10), tumour necrosis factor (TNF-*α*), triglycerides, cholesterol, fibrinogen, and plasminogen activator inhibitor-1 (PAI-1) [35]. 

Furthermore, selenium is an essential nutrient that plays an important role in bone status. That role is likely to involve the functions of selenoproteins. Many selenoproteins are antioxidant enzymes that participate in maintaining cell redox balance, which is important in the regulation of inflammation and bone cell proliferation/differentiation. Selenium (Se) may play additional cellular roles, particularly at supranutritional doses, that is, doses greater than those required for maximal selenoprotein expression. These include the induction of cell cycle arrest, apoptosis, immune function, and the prevention of the bone resorption through the inactivation of osteoclasts. These cellular activities of Se at both nutritional and supranutritional doses may partially account for the potential protection against rheumatoid arthritis, osteoarthritis, osteoporosis, and ROS produced by osteoclasts during bone remodeling [39, 40]. 

In a recent study [41], the authors have investigated that the effect of various carotenoids (carotene and xanthophylls including beta- (*β*-) cryptoxanthin, lutein, lycopene, b-carotene, astaxanthin, and rutin), and *β*-cryptoxanthin, which is abundant in Satsuma mandarin orange (*Citrus unshiu* Marc.), has a stimulatory effect on bone calcification *in vitro*. *β*-cryptoxanthin has stimulatory effects on osteoblastic bone formation and inhibitory effects on osteoclastic bone resorption *in vitro*, thereby increasing bone mass. *β*-cryptoxanthin has an effect on the gene expression of various proteins that are related osteoblastic bone formation and osteoclastic bone resorption *in vitro*. The intake of *β*-cryptoxanthin may have a preventive effect on bone loss in animal models for osteoporosis and in healthy human or postmenopausal women. In addition, the role of *β*-cryptoxanthin in bone health has been also shown in human subjects with epidemiological studies. The supplemental intake with the combination of *β*-cryptoxanthin and other nutritional factors may have a potential effect in the maintaining of bone health and decrease in bone loss [38].

### 4.1. Effect of *ω*-3 Fatty Acids on Bone Remodeling

Dietary fat may influence bone health. Long-chain polyunsaturated fatty acids (LCPUFAs), specially the omega-3 (*ω*-3) fatty acids such as EPA and DHA, are beneficial for bone metabolism. Several studies in humans have reported that LCPUFAs can increase bone formation, affecting peak bone mass in adolescents and reducing bone loss [14].

However, the cellular mechanisms of action of the LCPUFAs are complex and involve modulation of fatty acid metabolites such as prostaglandins, resolvins and protectins, cytokines, growth factors, and some other molecular signaling pathways. There are several mechanisms by which *ω*-3 PUFAs can regulate bone metabolism, including a decrease in the release of prostaglandin E2 (PGE2) and, in the most important osteoclast differentiation factor, receptor-activated nuclear kappa-*β* ligand (RANKL). Furthermore, *ω*-3 may modulate the number of proinflammatory cytokines, increasing production of IGF1 and improving calcium accretion in bone [14, 39].

The prostaglandins (PGs), produced from essential fatty acid precursors (20:4 *ω*-6 and 20:5 *ω*-3) in osteogenic cells, regulate both bone formation and bone resorption. In support of the relationships between dietary PUFA, PGs, and bone metabolism, Li et al. (1999) [40] reported that dietary lipids modulated *ex vivo *bone PGE2 production and the concentration of IGF-I in bone tissues and led to altered bone formation rates in growing chicks and rats. In these experiments, animals given long-chain *ω*-3 fatty acids tended to show an increased rate of bone formation, suggesting a stimulatory effect on osteoblastic activity. The favorable effect of *ω*-3 fatty acids on bone turnover in growing animals is supported by the observation of reduced bone mineral loss in ovariectomized rats supplemented with eicosapentaenoic acid (EPA 20:5 *ω*-3). The bone-sparing effect of 20:5 *ω*-3 may be associated with diminished bone resorption or increased bone formation. In addition, the action of *ω*-6 fatty acids (linoleic acid) with long-chain *ω*-3 fatty acids can benefit bone modeling [42]. 

**Figure 3:**
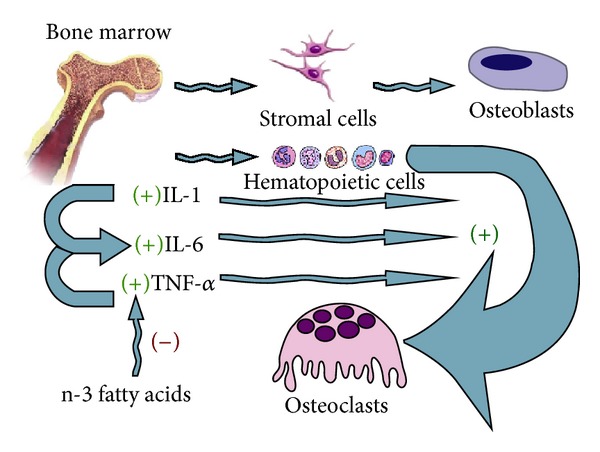
The relationship between cytokines and osteoclastogenesis. IL-1: interleukine-1; IL-6: interleukine-6; TNF-*α*: tumor necrosis factor-*α*.

In this sense, dietary intake of essential fatty acids plays a crucial role in the cell membrane and the production of various cytokines by inflammatory cells. The *ω*-6 fatty acids, especially arachidonic acid (AA), are the primary source of the *ω*-6 eicosanoids that are produced from the oxygenation of AA by cyclooxygenase, lipoxygenase, and epoxygenase enzymes to produce prostaglandins, leukotrienes, lipoxins, and P-450 compounds. The dietary long-chain *ω*-3 fatty acids eicosapentaenoic acid (EPA) and docosahexaenoic acid (DHA) partially replace the *ω*-6 fatty acids, particularly AA, in the membranes of platelets, erythrocytes, neutrophils, monocytes, and liver cells. This leads to a change in the ratio of *ω*-6/*ω*-3 fatty acids in membranes and a change in their function which can decrease the production of IL-1, IL-6, and TNF-*α*. The modulatory effect of essential fatty acids on cytokines plays a pivotal role in the pathogenesis of osteoporosis [43, 44] (Figure 3).

According to Casado-Díaz et al. [45] consumption of omega-3 may protect against osteoporosis because they may inhibit osteoclastogenesis. However, with aging, mesenchymal stem cells (MSC) in bone marrow are increasingly differentiated into adipocytes, reducing the number of osteoblasts. Products derived from omega-6 and omega-3 metabolism may affect MSC differentiation into osteoblasts and adipocytes. Indeed, MSC precursors may differentiate into adipocytes instead of into osteoblasts, in a process which is accelerated with aging. According to the ratio of omega-6/omega-3 consumed, certain types of metabolites will be produced, modifying some physiological processes and consequently the susceptibility or resistance to the appearance of different pathologies. In general, physiological processes involving metabolites derived from these two types of polyunsaturated fatty acids are in opposition to each other. So eicosanoids derived from omega-6 are considered mainly proinflammatory, while those derived from omega-3 are anti-inflammatory [45, 46]. 

The healthy recommended ratio of omega-6/omega-3 fatty acids is approximately 1 : 1; however actual estimations indicate that it is in fact much higher between 15 : 1 and 16.7 : 1. According to the evidence, a reduction in the omega-6/omega-3 proportion decreases the risk of suffering cardiovascular pathologies, tumors, and other chronic diseases, including osteoporosis [43]. It is hypothesized that these polyunsaturated fatty acids also act on bone formation, because the products of the metabolism of omega-6 and omega-3 fatty acids can act on precursor cells of osteoblast and adipocytes [45]. 

Various *in vivo* and *in vitro* studies have been carried out to study the possible incidence of omega-3 fatty acids in bone metabolism. Several lines of evidence have shown that the anti-inflammatory effect of omega-3 can lower the osteoclastic activity and thus reduce bone resorption [47].

A diet rich in omega-6 fatty acids which rises omega-6/omega-3 ratio seems to cause not only cardiovascular problems but also an increase in the adiposity of the bone marrow, by enhancing the adipogenic differentiation of MSC, inhibiting their differentiation into osteoblasts. Consequently, this could have a negative impact on bone metabolism. Furthermore, a diet with a suitable proportion of omega-6/omega-3 fatty acids appears to avoid pathologies in the bone health associated with aging, such as osteoporosis [43]. This is because omega-3 fatty acids do not exert a strong adipogenesis induction capacity as the one of the omega-6 fatty acids, thus allowing the osteoblastogenesis. That fact, together with their inhibitory effect on osteoclastogenesis, may improve the maintenance of the bone mineral mass.

The NHANES III data has established the correlation between dietary fat and hip BMD (bone mineral density) in 14,850 men and women. There was no significant association between total fat intake and BMD at any sites of the analysis. On the other hand, saturated fat intake was negatively associated with BMD in the femoral neck across all subjects, and the greatest effect was observed in men younger than 50 years of age. This indicates the probability of more vulnerability of men to the undesirable effects of saturated fat on bone density [48]. This study is in agreement with another study which was conducted with 20 men and 3 women using a controlled feeding protocol. Significant reduction was reported in N-telopeptide (NTx) (a key marker of bone resorption) when the subjects consumed diet containing ~8% saturated fatty acid (SFAs) and ~17% PUFAs compared with a diet containing ~13% SFAs and ~9% PUFAs [49].

In other research, it was investigated the role of fatty acids on bone accumulation and attainment of peak bone mass in young men. In this cohort study 78 healthy young men were enrolled. BMD of the total body, hip, and spine was measured at baseline and at 22 and 24 years of age. Fatty-acid concentration was measured at 22 years of age. The results showed that *ω*-3 fatty acids, especially DHA, are positively associated with bone mineral accumulation and therefore with peak BMD in young men [50]. In the first controlled feeding study in humans, the effect of dietary plant-derived *ω*-3 PUFAs on bone turnover was assessed. In this study, 23 subjects consumed each diet in a randomized, three-period crossover design. Each diet consisted of different amounts of saturated and unsaturated fatty acids. Bone turnover was assessed by the differences in serum concentrations of NTx and BSAP at baseline and six weeks later. There was no change in the levels of BSAP across the three groups. The NTx levels showed significant changes and positively correlated with the proinflammatory cytokine TNF-*α* in all the groups. The authors concluded that plant sources of dietary *ω*-3 PUFAs may have a protective effect on bone metabolism via a decrease in bone resorption in the presence of consistent levels of bone formation [49, 51].

#### 4.1.1. *ω*-3 Fatty Acids and Oxidative Stress

It is well known that reactive oxygen species (ROS) and oxidative stress are one of the main factors in the regulation of bone status [52]. The role of ROS in bone metabolism is unique and dual due to their effect under physiological and pathological conditions. Under physiological conditions, the production of ROS by osteoclasts assists in accelerating destruction of calcified tissue and hence assists in bone remodeling [53, 54].

Although enhanced osteoclastic activity and increased production of ROS are linked in many skeletal pathologies, it remains to be unclear whether increased ROS production overwhelms the antioxidant defenses, in subjects with oxidative stress.

Osteoclasts chisel away at older bone, opening tiny holes that will be refilled by osteoblasts. The chisel molecule is a superoxide radical, which is one of the highest reactive oxygen species (ROS). ROS also play a specific role in sculpting or remodeling of bone. The role of antioxidants in controlling ROS formation is also well established. In this way, decreased activity of the antioxidant enzymes, SOD, and GSH-PX showed a defense mechanism that appears to have been overtaken in reducing the increased superoxide production by the osteoclasts [53]. 

A long-term study reported that *ω*-3 FA reduce peroxidation of lipids (reducing oxidative stress) [55]. Acute enrichment of plasma with *ω*-3 FA did not increase lipid-mediated insulin resistance; neither was oxidative stress reduced, therefore, an inhibitory effect on FA oxidation is possible.

The influence of *ω*-3 fatty acids on lipid peroxidation *in vivo* depends on the balance of oxidative stress and induced antioxidative enzymes, but the overall effect appears contradictory. This is probably due to the different doses of *ω*-3 fatty acids used, different amounts of antioxidants supplied, and different methods used to quantify the oxidative stress in previous studies [56]. Thereby, when the level of F2-isoprostane was used as a criterion, all studies demonstrated no enhancement of *in vivo* oxidant stress after *ω*-3 fatty acid supplementation [57, 58].

The susceptibility of fatty acids to oxidation is known to be directly dependent on their degree of unsaturation. Nevertheless, some *in vitro* [59] and *in vivo* [58, 60] studies suggest that the relation between chemical structure and susceptibility to oxidation is not as straightforward as hypothesized from theoretical viewpoints. Some evidence indicates that kinetics of fatty acid oxidation depend on the compartment in which they react with oxidants: aqueous environments (such as those at the cell membrane/plasma and cytosol/cell membrane interfaces) yield different oxidation profiles than the organic ones [61].

Based on evidence, the higher number of unsaturations present in *ω*-3 long-chain fatty acids has been postulated to enhance lipid peroxidation. However, the influence of fish oil supplementation on some indicators of oxidative stress was not consistent, partly because *ω*-3 fatty acids also enhanced the activities and expression of antioxidant enzymes, such as catalase, glutathione peroxidase, and superoxide dismutase [56, 62]. For example, when individual fatty acids are oxidized in an aqueous environment, the generation of peroxidation products does not reflect their degree of unsaturation [63]. Furthermore, Mazière et al. (1998) [61] compared the effects of omega-6 and omega-3 fatty acids, incorporated into endothelial cells, with respect to cellular ability to oxidize LDL. They reported that omega-3 fatty acids lowered TBARS production, superoxide anion secretion, and LDL peroxidation compared with omega-6. Their interpretation was that omega-3 was less efficiently incorporated into cellular lipids, even though after a similar percentage of PUFA incorporation, omega-3 fatty acids still induced a less marked increase in LDL modification as compared with omega-6. One explanation proposed by the authors was that the omega-3 series, due to the position of their unsaturations, is less susceptible to oxidative damage than the omega-6 series [63]. The peroxide tone theory [64] also contributes to explain the inhibitory effects of omega-3 fatty acids on the vascular production of inflammatory molecules, namely, eicosanoids (via both COX isoforms inhibition), cytokines [65], and leukocyte adhesion molecules. 

From a mechanistic viewpoint, NAD(P)H oxidase is one of the major contributors to endothelial free radical production: its inhibition by DHA and (presumably) other PUFAs might greatly explain the observed effects on ROS production. Massaro et al. (2006) [66] also reported DHA-mediated inhibition of IL-1a-induced ROS production, which would contribute to the anti-inflammatory actions of omega-3 fatty acids at the endothelial level. Other potential mechanisms of action of LCPUFAs would be that they act as a “sink” to trap free radicals, hence becoming oxidized themselves [63].

*In vivo* situations induce changes in the relative distribution, and proportion of PUFAs in cell membranes and lipoproteins may result in modifications of their susceptibility to oxidation, whose extent cannot be easily predicted on the sole basis of their fatty acid composition. Furthermore, PUFAs, especially those of the omega-3 series, are precursors of anti-inflammatory lipid mediators such as protectins, resolvins, and neuroprotectins [63, 67].

In conclusion, based on evidence showing reduced excretion of lipid peroxidation products, that is, isoprostanes, after polyunsaturated fatty acid intake and data on ROS production and direct superoxide removal by LCPUFAs, mainly those of the omega-3 series, this series of fatty acid might indirectly act as anti- rather than prooxidant in vascular endothelial cells, thereby decreasing inflammation and, in turn, the risk of atherosclerosis [63]. 

#### 4.1.2. *ω*-3 Fatty Acids and Inflammatory Signaling

The inflammatory response clears and fights against infection, repairing damaged tissue and organ systems, and removing harmful chemical. Though the net process is protective, the failure to resolve the inflammation and return the target tissue to homeostasis can result in disease. Evidence has provided that dietary *ω*-3 polyunsaturated fatty acids and eicosapentaenoic (EPA, 20:5 *ω*-3) and docosahexaenoic acid (DHA, 22:6 *ω*-3) in particular are important modulators of inflammatory responses [68].

Dietary intake of *ω*-3 PUFAs is known to be associated with the decrease of oxidative and inflammatory markers, whereas a high *ω*-6/*ω*-3 PUFA ratio is associated with increase of inflammation [69]. The fact indicates that PUFAs might regulate systemic inflammation status.

The metabolites of PUFAs actively participate in cell signal transduction pathways and to some degree regulate gene expression. Generally, *ω*-3 PUFAs produce 3-series prostaglandins and 5-series leukotrienes by cyclooxygenase-1 (COX-1) [47]. Increased consumption of *ω*-3 PUFAs could consequently inhibit *ω*-6 PUFA derived prostaglandin synthesis from COX-2. Therefore inhibition of inflammatory responses, such as COX-2 induction and prostaglandin E2 synthesis, may be effectively involved in the effect of *ω*-3 PUFAs and in the difference between *ω*-3 and *ω*-6 PUFAs [70].

The fact that selective COX-2 inhibitors gave satisfactory relief of symptoms in both osteoarthritis and rheumatoid patients suggests that eicosanoids participate in the inflammatory process of these severe bone/joint diseases. COX-2 and its product PGE2 appear to be a common link between the two disease states, and since it is possible to regulate the activity and expression of COX-2, this enzyme is a potential target for dietary intervention in optimizing bone formation and controlling bone disease [71]. 

Inflammatory cytokines (e.g., IL-1, a major player in rheumatoid arthritis (RA) and osteoarthritis (OA)) are known to inhibit chondrocyte proliferation and induce cartilage degradation, therefore part of the response may be mediated by PGE2. Excessive production of PGE2 is linked to joint pathology (rheumatoid arthritis), known to exacerbate inflammatory responses, and results in a net loss of proteoglycan from articular cartilage [71]. *ω*-3 PUFA dietary supplements ranging from 3 to 6 g/day showed a modest, but rather consistent beneficial effect of these fatty acids in joint disease. In the same way, the syntheses of proinflammatory factors IL-1, IL-2, and TNF-*α* in cartilage tissue were suppressed by dietary supplementation with fish oil containing both EPA and DHA [71, 72].

The mode of *ω*-3 fatty acid action in RA patients could be related to eicosanoid biosynthesis. One explanation for this phenomenon is that the EPA metabolite PGE3 has a higher proinflammatory effect compared with PGE2 [73]. Lowering PGE2 in the diseased joint with diets rich in long-chain *ω*-3 fatty acids could further benefit RA patients by reducing bone resorption since PGE2 stimulates osteoclast activity, which results in secondary osteoporosis. Since PGE2 activation of the IGF-I/IGFBP axis may play an important role in cartilage biology and collagen and proteoglycan synthesis, dietary fatty acids may also be important for supporting joint repair. Further investigations are needed to describe the effects of nutraceutical fatty acids and the ratio of *ω*-6/*ω*-3 fatty acids on cartilage biology, joint disease, and ligament healing since dietary sources of these fatty acids exert potent effects on prostanoid biosynthesis in controlling cell activity in these processes [71].

It has been suggested that dietary supplementation with LC *ω*-3 PUFA can suppress the production of TNF-*α* [73], but no significant changes in IL-6 and TNF-*α* were observed in a study when subjects received either 2 g, 4 g, or 6 g per day of Hi DHA (26% DHA and 6% EPA) over 12 weeks [74]. Similar findings were reported in a study that did not observe any effect on levels of IL-6 concentrations after supplementation with 3.4 g per day of LC *ω*-3 PUFA for 6 weeks. Another study reported that weight loss but not LC *ω*-3 PUFA influenced a significant reduction in IL-6 and a nonsignificant reduction in TNF-*α*. These authors suggest that as IL-6 is secreted by adipose tissue, a reduction in fat mass could contribute to a reduction of IL-6 levels [75]. 

It has also been reported that DHA has more potent anti-inflammatory effects than EPA, and that DHA is a key controller of hepatic lipid metabolism and is ultimately involved in the suppression of lipogenesis which could impact on weight management. In this way, there are gender difference in the metabolism of LC *ω*-3 PUFA with studies showing significantly higher concentrations of DHA in females compared to males [76, 77].

There is conflicting evidence on the effect of LC *ω*-3 PUFA on plasma leptin levels, with some studies reporting that EPA increases leptin levels [66], and others suggesting that LC *ω*-3 PUFA reduces leptin levels [78]. 

Madsen et al. (2008) [79] reported an increase in levels of adiponectin in response to diet induced weight loss but suggest that a reduction in weight of at least 10% is required to show a signficant increase in levels of adiponectin.

Conflicting findings have also been reported from studies investigating the effect of diet and/or weight on C-reactive protein (CRP) levels. One study reported that LC *ω*-3 PUFA but not weight loss was associated with a significant reduction in CRP [80], while another study reported an inverse association with LC *ω*-3 PUFA and serum CRP in men [81]. Observational studies support that *ω*-3 is associated with lower levels of CRP [82], while randomised control trials have reported no effect of LC *ω*-3 PUFA on CRP levels in healthy people [83]. It has also been suggested that a reduction in the level of CRP is directly related to a reduction in weight. However, a significant reduction in the long term requires a weight loss greater than 10%, and not only differences in the length of the intervention but also differences in ages of subjects within the groups and components of the different diets could influence levels of plasma CRP [73]. 

#### 4.1.3. *ω*-3 Fatty Acids and Obesity

Obesity is regulated by cytokines and endocrine factors that have effects on bone and calcium absorption; obesity also increases bone fracture risk. Long-chain omega-3 polyunsaturated fatty acids (LC *ω*-3 PUFA) are essential nutrients that play a beneficial role in several pathological processes because of their anti-inflammatory effects, calcium absorption, and modulation of lipids. Omega-3 fatty acids, EPA and DHA predominantly, have shown preliminary results in animal and human studies in the treatment and prevention of obesity. 

There is controversy about the beneficial effects of supplementation with LC *ω*-3 PUFA on reducing adiposity. Some studies have associated *ω*-3 PUFA with weight loss [84], in others with a decrease in adipose tissue mass [85], whereas other studies have shown no effect on adiposity [86]. It has also been suggested that concentrations of *ω*-3 are significantly lower in obese compared to normal weight adults, adolescents, and children suggesting that high concentrations of *ω*-3 in the body inhibit the development of adiposity or have possibly reduced weight previously gained. In this respect, if obese individuals are given time to increase levels of *ω*-3 prior to commencing a reduced energy weight loss diet, it may result in greater weight reduction [87].

A growing evidence supports that there are gender differences in the metabolism of *ω*-3 PUFA. It has been reported that not only higher levels of DHA and lower levels of EPA are circulating in serum lipids in females compared to males, but also females are more responsive to the metabolism of LC *ω*-3 PUFA compared to males. Other studies have also reported that females had a higher percentage of total fatty acids as DHA in plasma and adipose lipids compared to males [87].

The mechanisms by which LC *ω*-3 PUFA assist the reduction of body fat and/or body weight are still being explored. It has been suggested that *ω*-3 modulates lipid metabolism promoting lipolysis and enhancing hepatic fatty acid oxidation, inhibiting fatty acid synthesis and VLDL (very low density lipoprotein) secretion. It has been established that DHA is a key controller of hepatic lipid synthesis, has a major impact on hepatic lipid metabolism, and is ultimately involved in the suppression of lipogenesis [88]. 

It is possible that an extended period of *ω*-3 intake during the growth years rather than dietary supplementation with large doses over a short duration in adults influences body weight. In this context, several studies have reported a significantly higher concentration of *ω*-3 in normal weight compared to obese individuals but timing and duration of intake of *ω*-3 in the individuals is unknown [89].

It is also possible that incorporation of *ω*-3 LCPUFA into the adipose tissue is needed for any effects on weight loss to occur and, despite long-term supplementation in adults, an increase in the levels of EPA and DHA in adipose tissue is reportedly modest [86]. As late foetal and early postnatal life is a highly sensitive period when adipose tissue expands rapidly [90], this could be a critical opportunity to improve the balance of fatty acids involved in adipogenesis and lipogenesis. A study in humans, where mothers received DHA supplementation from 21 weeks gestation until the end of the third month of lactation, reported a significant time-dependent effect of DHA on weight and body mass index (BMI) reduction in their infants at 21 months [91]. Thus, it would appear that when increasing levels of *ω*-3 for weight management, gender, age, duration of supplementation, concentration, and ratio of EPA : DHA all require further consideration. 

While there is evidence from a considerable number of animal studies that LC *ω*-3 PUFA can reduce body fat, their impact on body composition in humans is less evident due to a lack of appropriately designed studies of adequate size and duration [92].

In human studies it is becoming apparent that an increased intake of LC *ω*-3 PUFA by 0.3–3.0 g/day can reduce body weight and body fat in overweight and obese individuals. The mechanism by which increasing the intake of *ω*-3 PUFA by such an amount may improve body composition is most likely altered gene expression favoring increased fat oxidation in adipose and skeletal muscle tissue and reduced fat deposition in adipose tissue. Some studies have reported that an increase in *ω*-3 PUFA intake within this dose range might also attenuate postprandial sensations of hunger, although the majority of studies which have reported improved body composition have not reported any reduction in food (i.e., energy) intake. Data from other studies also support that LC *ω*-3 PUFA might promote increases in lean tissue mass, thus potentially increasing metabolic rate and indirectly assisting with body fat reduction. It might be possible that the increases in vasodilator function and muscle blood flow during exercise resulting from supplementation with 1.9–5.0 g/day of LC *ω*-3 PUFA might promote nutrient disposal by skeletal muscle, thus reducing the availability of nutrients for lipogenesis and storage in adipose tissue [92]. 

*ω*-3 PUFA have been shown to affect body composition and to reduce the accumulation of body fat, thereby affecting body weight homeostasis. However, in adults, the studies analyzed [93–95] show no change in body weight by dietary supplementation with *ω*-3 PUFA, except for one [96]. The results do not provide data strong enough to conclude that *ω*-3 PUFA can modify and particularly reduce body weight. Marked differences in experimental design, intervention type and duration, baseline characteristics of the participants (degree of obesity, associated condition, etc.), attrition rate, dose of *ω*-3 PUFA, and EPA/DHA ratio make the results inconclusive and, in some cases, discordant [97]. 

An observational study of 124 adults found that obese individuals had significantly lower plasma *ω*-3 PUFA concentration compared to healthy weight participants. In obese subjects, there was a significant inverse correlation between plasma *ω*-3 PUFA and BMI [98].

Studies in youth report significantly decreased plasma *ω*-3 PUFA concentration in overweight compared to healthy youth; in obese youth plasma *ω*-3 PUFA is significantly inversely related to BMI [98].

Randomized controlled trials in humans examining the relationship between *ω*-3 supplementation and body composition have found conflicting results. This may be due to differences in study design, the dosage, timing, and duration of *ω*-3 PUFA administration, and the use of other supplements in addition to *ω*-3 PUFA [99].

Published literature supports evidence for a role of *ω*-3 PUFAs in body composition. A study of 2-month *ω*-3 PUFA supplementation in 26 overweight or obese postmenopausal women with diabetes found a reduction in body fat mass and a reduction in adipocyte diameter, though no reduction in body weight or total energy intake was observed [94]. An 8-week study of 278 overweight adults found that those on a restricted caloric diet rich in lean or fatty fish or fish oil had a significant reduction in waist circumference and weight compared to individuals on a calorie restricted diet, but this effect was only seen in men [100]. Participants in this study on the high *ω*-3 PUFA diets reported more fullness immediately after a test meal and more fullness and less hunger 2 hours postprandial than those on a low *ω*-3 PUFA diet [101]. This finding provides a potential role for *ω*-3 in appetite regulation in humans.

Moreover, different organ systems in the body and various pathways are involved in appetite, food intake, and energy homeostasis, and the disruption of these systems leads to obesity. These include brain structures such as the brain stem, hypothalamus, and reward pathways, as well as the gastrointestinal tract, adipose tissue, and the pancreas. Increasing evidence suggests that the *ω*-3 fatty acids EPA and DHA play an important role in these organ systems and especially in the central nervous system [99]. 

While the effects of EPA/DHA on the endocannabinoid system and on dopaminergic reward systems in the brain have been described, no animal or human studies have examined the role of DHA and EPA in modulating these systems to affect appetite and food intake. As the endocannabinoid and mesocorticolimbic pathways play a role in appetite, energy intake, and obesity, we hypothesize that, in addition to beneficial effects on metabolism, EPA and DHA regulate the endocannabinoid and mesocorticolimbic dopamine systems in humans to decrease appetite, increase satiety, reduce food intake, and ultimately contribute to prevent or reduce overweight and obesity [99]. 

Further studies are necessary to elucidate the effects of EPA/DHA supplementation on the reward associated with food intake and appetite, food consumption, weight loss, and, at the same time, the molecular effects on the function of the endocannabinoid system and the mesocorticolimbic system. Studies in humans are especially noteworthy, because molecular and behavioral changes in animal models may not correspond to the same effects in humans. In addition, the interplay between diets of differing composition of essential fatty acids and effects of EPA/DHA must be further examined because the effects of *ω*-3 PUFAs also depend on the ratio of *ω*-3 to *ω*-6 fatty acids, as they are substrates that compete for some of the same enzymes [99].

Animal and human studies indicate that EPA and DHA supplementation may be protective against obesity and might reduce weight gain in already obese animals and humans. There is evidence in animal studies that *ω*-3 PUFA supplementation can modulate fat deposition, food intake, and body weight. Nevertheless, we should use caution when making inferences to the effects of *ω*-3 PUFA in humans, because of possible differences of EPA and DHA supplementation between animals and humans and because the doses used in animal studies vary widely and are typically higher than those considered safe in humans [99]. 

#### 4.1.4. *ω*-3 Fatty Acids in Menopause and Aging

The best way to avoid osteoporosis in women is to build strong bones in early life by consuming a well-balanced diet (vitamin D, calcium, *ω*-6 and *ω*-3 fatty acids, etc.) and to follow a routine exercise program pre- and postmenopause. 

Although osteoporosis occurs in both women and men, postmenopausal women have the greatest risk of developing this disease. Extensive research has been conducted to find a cure for the disease; however, optimizing bone development in the young and reducing bone resorption to maintain bone mass and restore skeletal integrity in the older are still the best means to control the disease. Direct evidence of any beneficial effect of dietary *ω*-3 fatty acids on human osteoporosis is still lacking. However, experiments using animal and cell culture models and epidemiological data suggest promising applications of *ω*-3 PUFA on this disease [102].

Some studies suggest that using *ω*-3 fatty acid supplements, which are antagonistic to arachidonic acid (AA) in the sense of eicosanoid action, could help maintain bone mineral content after menopause in women. Considering the results from these ovariectomized rat studies (inhibitory to bone resorption) and the findings on *ω*3 fatty acids in bone modeling (promoting bone formation), it is plausible that consuming diets rich in *ω*-3 fatty acids will help to build and maintain a healthy skeleton in the human [102, 103].

Högström et al. (2007) [50] reported that serum phospholipid levels of *ω*-3 PUFA and DHA were positively associated with bone mass density (BMD) in healthy young men. However, there has been no study investigating the relationship between tissue fatty acid composition and BMD in women, and studies on the effect of *ω*-3 PUFA on bone health are inconclusive.

There is an available study that demonstrates that tissue levels of *ω*-3 PUFA are positively correlated with bone mass in postmenopausal women. High erythrocyte levels of EPA + DHA reduced the risk of osteoporosis in postmenopausal Korean women [104].

Epidemiologic studies have shown a positive association between BMD and the intake of n-3 PUFA in older adults or the intake of fish in postmenopausal women and the elderly [105]. A cross-sectional study in China also reported a greater bone mass and lower risk of osteoporosis among postmenopausal women with a higher intake of sea fish [106]. In addition, clinical trials by Tartibian et al. demonstrated that 1 g/day of *ω*-3 PUFA supplementation over a period of 24 weeks was effective in reducing chronic inflammation and increasing BMD in postmenopausal women [107]. 

Although the mechanism is not known, *ω*-3 PUFA has been shown to reduce the formation of prostaglandin E2, which stimulates bone formation through increased production of an insulin-like growth factor, a powerful growth stimulator for bone and muscle 5. *ω*-3 PUFA has also been shown to increase calcium absorption and decrease the production of inflammatory cytokines induced by osteoclastogenesis [104]. 

In the female population, postmenopausal individuals are regarded as more vulnerable to oxidative stress (OS) than those in reproductive age, because their oxidative balance is deranged not only by the generally older age but also by a lower level of 17*β*-estradiol (E2), believed to act as an antioxidant. Consistently, a considerable amount of *in vitro* evidence suggests that ROS could be involved in the pathogenesis postmenopausal osteoporosis (PO) which is characterized by increased bone loss and consequent higher risk of fractures [108]. Further experiments on human osteoclast like cell lines showing that E2 enhances the intracellular antioxidant defences suggest that estrogenic skeletal protection occurring in premenopausal period of female could be attributed, at least partly, to the opposing activity of these hormones toward OS development [109].

OS is not itself a characteristic condition of subjects affected by PO, but that it might influence BMD loss in women who have already experienced the menopausal transition, thus increasing the risk of this severe bone degenerative disease. In other terms it can, therefore, be assumed that the association between altered hydroperoxide levels and the selective decline in BMD in postmenopausal women might reflect an interplay between OS and the menopausal endocrine changes [109]. 

Although relevant progress has been achieved in the understanding of how estrogen deficiency induces bone loss, the underlying pathogenic mechanisms have proven to be remarkably complex. One of the mechanisms suggested takes into account the ability of E2 to regulate receptor activator of nuclear factor-kappa *β* (NF-*κβ*) ligand- (RANKL-) RANK-osteoprotegerin pathway. RANK along with OS is, in turn, potent activators of NF-*κβ*, the osteoclastogenic factor that regulates osteoclast differentiation and thus bone resorption and remodelling [108].

Pansini et al. (2008) [110] does not show any significant correlation between menopause, level of circulating E2, and markers of OS. Indeed, if E2 has an antioxidant action against level of systemic oxidative damage (as in the *in vitro* inhibition of LDL oxidation), this is usually obtained only at supraphysiological levels of hormone. 

The involvement of oxidative status in the relationship between estrogen PO is further supported by the finding that in osteoclast cell lines estrogen upregulate glutathione peroxidase responsible for the intracellular degradation of hydrogen peroxide and major antioxidant enzymes of these cells [109].

Sharif et al. (2010) [111] demonstrated no significant effect of *ω*-3 fatty acids on bone formation markers (osteocalcin and alkaline phosphatase) in osteoporotic women; it showed significant beneficial effects of n-fatty acids on bone resorption marker (pyridinoline).

*ω*-3 PUFA may play various roles, possibly affecting bone formation and resorption through calcium absorption and production of prostaglandins and cytokines. Epidemiological studies suggest that the intake of fish or n-3 PUFA is associated with greater bone mass in both women and men. Additionally, in clinical trials, supplementation with fish oil or EPA has been shown to increase bone mineral density (BMD) in elderly patients and postmenopausal women.

A cohort study that evaluated the effects of omega-3 fatty acids on bone health has investigated the associations between dietary polyunsaturated fatty acid and fish intakes and hip bone mineral density (BMD) at baseline (*n* = 854) and changes 4 years later in individuals (*n* = 623) with a mean age of 75 years in the Framingham Osteoporosis. High intakes (>3 servings/wk) of fish relative to lower intakes were associated with maintenance of femoral neck bone mass density (FN-BMD) in men (dark fish + tuna, dark fish, and tuna) and in women (dark fish), thus suggesting that fish consumption may protect against bone loss [105]. 

## 5. Conclusion

According to the physiological mechanisms of *ω*-3 fatty acids and available data on human studies, the consumption of *ω*-3 fatty acids might be beneficial on bone metabolism. Most studies have been conducted in women, but recent epidemiological data suggest that the effects of dietary fats on bone health may be particularly strong in men. Some studies report that bone integrity in both men and women may benefit from manipulation of the dietary fatty acid profile, especially the dietary ratio of *ω*-6/*ω*-3 fatty acids. Controlled feeding trials are needed in order to demonstrate convincingly a causal relationship between fatty acids and bone health, the optimal dose of *ω*-3 fatty acids, and the optimal ratio of *ω*-6/*ω*-3 fatty acids. Lipid intake may be targets for the development of *ω*-3 PUFA containing dietary bioactive agents to down-modulate inflammatory and immune responses and for the treatment of autoimmune and chronic inflammatory diseases. Regarding the pathogenesis of osteoporosis and the interaction between various cells and mediators, many studies have been conducted to evaluate the benefit of different therapeutic options, therefore evidence has been growing on the effects of dietary fatty acids on bone health. 

There is growing evidence that LC *ω*-3 PUFA can improve body composition in humans, but some aspects are still controversial, because the majority of studies have been of relatively short duration and the magnitude of the improvements have been modest. Accordingly, there is a need for long-term studies to determine the relative effects of EPA and DHA supplementation on body composition and the feasibility of using LC *ω*-3 PUFA supplementation as a strategy to improve body composition in overweight and obese populations. In several animal studies, *ω*-3 PUFA supplementation can modulate fat deposition, food intake, and body weight. However, we should use caution when making inferences to the effects of *ω*-3 PUFA in humans, because of possible differences in pharmacokinetics of EPA and DHA supplementation between animals and humans and because the doses used in animal studies vary widely and are typically higher than those considered safe in humans. Although evidence for a role of *ω*-3 fatty acids in prevention of overweight and obesity is just beginning to be accumulated, the various other health benefits and lack of negative side effects warrant consideration of the need to encourage dietary changes to increase *ω*-3 PUFA or the use of *ω*-3 PUFA supplements at the population level. Even if the effect of *ω*-3 PUFA on overweight and obesity is found to be small, such changes at the level of the individual can lead to significant shifts in the distribution of weight in the population. 

In conclusion, strong evidences featured in the scientific literature available to date support the benefits of *ω*-3 fatty acids in bone health. Several mechanisms have been proposed (affecting bone formation, bone resorption, serum calcium and vitamin D, oxidative stress, and inflammatory mediators). However, neither the exact benefit nor the exact mechanism of action of essential fatty acids has been determined yet. Therefore, although there are some clinical evidences linking bone metabolism and dietary lipids, more clinical trials are necessary to prove whether dietary changes or *ω*-3 PUFA supplementation play a major role in bone health and turnover.
